# Morbidity and Mortality Associated with Typhoid Fever Among Hospitalized Patients in Hyderabad District, Pakistan, 2017-2018: Retrospective Record Review

**DOI:** 10.2196/27268

**Published:** 2021-05-17

**Authors:** Munaza Fatima, Santosh Kumar, Mudassar Hussain, Naveed Masood Memon, Anum Vighio, Muhammad Asif Syed, Ambreen Chaudhry, Zakir Hussain, Zeeshan Iqbal Baig, Mirza Amir Baig, Rana Jawad Asghar, Aamer Ikram, Yousef Khader

**Affiliations:** 1 Field Epidemiology and Laboratory Training Program Islamabad Pakistan; 2 National Institute of Health Pakistan Islamabad Pakistan; 3 Global Health Strategists and Implementers Islamabad Pakistan; 4 Department of Public Health, Community Medicine and Family Medicine Faculty of Medicine Jordan University of Science and Technology Irbid Jordan

**Keywords:** antimicrobial resistance, complications, control drug resistance, extensive drug resistance, hospitalization, Hyderabad, ileal perforation, medical records, microbiological, morbidity, mortality, Pakistan, prevention, typhoid

## Abstract

**Background:**

Hyderabad, Pakistan, was the first city to witness an outbreak of extensively drug resistant (XDR) typhoid fever. The outbreak strain is resistant to ampicillin, chloramphenicol, trimethoprim-sulfamethoxazole, fluoroquinolones, and third-generation cephalosporin, thus greatly limiting treatment options. However, despite over 5000 documented cases, information on mortality and morbidity has been limited.

**Objective:**

To address the existing knowledge gap, this study aimed to assess the morbidity and mortality associated with XDR and non-XDR *Salmonella* serovar Typhi infections in Pakistan.

**Methods:**

We reviewed the medical records of culture-confirmed typhoid cases in 5 hospitals in Hyderabad from October 1, 2016, to September 30, 2018. We recorded data on age, gender, onset of fever, physical examination, serological and microbiological test results, treatment before and during hospitalization, duration of hospitalization, complications, and deaths.

**Results:**

A total of 1452 culture-confirmed typhoid cases, including 947 (66%) XDR typhoid cases and 505 (34%) non-XDR typhoid cases, were identified. Overall, ≥1 complications were reported in 360 (38%) patients with XDR typhoid and 89 (18%) patients with non-XDR typhoid (*P*<.001). Ileal perforation was the most commonly reported complication in both patients with XDR typhoid (n=210, 23%) and patients with non-XDR typhoid (n=71, 14%) (*P*<.001). Overall, mortality was documented among 17 (1.8%) patients with XDR *S* Typhi infections and 3 (0.6%) patients with non-XDR *S* Typhi infections (*P=*.06).

**Conclusions:**

As this first XDR typhoid outbreak continues to spread, the increased duration of illness before hospitalization and increased rate of complications have important implications for clinical care and medical costs and heighten the importance of prevention and control measures.

## Introduction

Typhoid fever is caused by *Salmonella enterica* serovar Typhi (hereinafter referred to as “Typhi”) and is characterized by prolonged fever, headache, nausea, loss of appetite, constipation, or diarrhea [1]. Globally, South Asia accounts for more typhoid fever cases each year than any other geographic region [2,3]. An estimated 11-21 million cases of typhoid fever and approximately 128,000-161,000 deaths are reported annually [3]. Among countries in South Asia, Pakistan has the highest estimated incidence rate of typhoid fever (493.5 per 100,000 persons/year) [4,5]. Treatment of enteric fever has been complicated by the development and rapid global spread of typhoidal organisms resistant to ampicillin, trimethoprim-sulfamethoxazole, and chloramphenicol. Additionally, the development of increasing resistance to fluoroquinolones is a growing challenge [6]. Since the extensively drug resistant (XDR) Typhi outbreak began in Hyderabad in late 2016 [7], typhoid fever cases in Pakistan increased, particularly in Hyderabad and other parts of Sindh province; this is attributed to the circulating XDR Typhi strain [8].

The most common serious and fatal complication of typhoid fever is intestinal perforation [9]. Even though many advancements have been made in patient care, morbidity and mortality rates remain high among patients with typhoid perforation, especially in low- and middle-income countries [10,11]. The high incidence of typhoid ileal perforations in developing countries is reportedly associated with delayed diagnosis and treatment and the emergence of antibiotic resistance. Once complications appear, patients require hospitalization and sometimes tertiary care [12]. Postoperative complications and mortality are common [13].

In November 2016, a multidrug-resistant Typhi strain that is resistant to ampicillin, chloramphenicol, and trimethoprim-sulfamethoxazole and additionally to ciprofloxacin and ceftriaxone was identified in patients hospitalized in Hyderabad, Pakistan. This XDR Typhi strain belongs to the H58 lineage and includes a plasmid-mediated blaCTX-M-15 extended-spectrum β-lactamase [14]. Typhoid cases have since been identified in Hyderabad, Karachi, and in other districts of Sindh province, and over 5372 cases were reported by December 2018. As of that month, no isolates resistant to azithromycin or a carbapenem had been reported. Despite the large number of cases, little information on mortality and morbidity attributable to XDR Typhi infections has been available. To address this knowledge gap, this study aimed to assess the morbidity and mortality associated with XDR and non-XDR Typhi infections in Hyderabad, Pakistan.

## Methods

The medical records of all hospitalized patients with culture-confirmed typhoid fever between October 1, 2016, and September 30, 2018, at the 5 major hospitals in Hyderabad, including Liaquat University City, Jamshoro, Shah Bhittai, Qasimabad, and Peratabad Hospitals, were reviewed. Subsequently, the pediatric, medical, and surgical ward records of all identified cases were also reviewed. We obtained ethics approval from the institutional review board of Liaqat University of Medical Sciences, Jamshoro, and administrative approvals from all the participating hospitals.

We collected data on demographic characteristics, date of fever onset, illness duration and antibiotic history before hospitalization, physical examination findings, results of serological and microbiologic tests, treatments prescribed or administered in hospital, date of recovery, duration of hospitalization, complications (including ileal perforation, septicemia, cholecystitis, pneumonia, jaundice, encephalopathy, meningitis, and deaths), and antimicrobial susceptibility of Typhi isolates. XDR Typhi cases were defined as those whose isolates were resistant to the 5 aforementioned agents. Ileal perforations were diagnosed if biopsy reports indicated “a hollow viscous perforation of the terminal part of the ileal wall” and pus culture sensitivity reports indicated an XDR status among patients with ileal perforation.

Data were recorded using a specially designed questionnaire and transferred to a computerized database for statistical analysis in Epi Info (Centers for Disease Control and Prevention). The data are expressed as mean, median, and percentage values. The chi-square test was used to compare percentages and the Mann–Whitney *U* test was used to compare medians. A *P* value of <.05 was considered significant.

## Results

A total of 1452 cases of culture-confirmed typhoid fever were identified from in-patient departments of 5 hospitals in Hyderabad during the study period. Patient ages ranged 1-80 (mean 14, median 22) years. More than half (n=791, 55%) of the patients were aged between 2 and 10 years. Almost two-thirds (n=915, 63%) of patients were male. The total number of monthly cases ranged from 2 in October 2016 to 98 in May 2018. Typhi isolates from approximately two-thirds of cases (n=947, 66%) were XDR, while the remainder (n=505, 35%) were non-XDR. Patients with non-XDR typhoid belonged to a slightly younger age group. Males accounted for similar proportions of XDR and non-XDR cases (64% and 62%, respectively) (Table 1).

The signs and symptoms in patients with XDR typhoid and those with non-XDR typhoid on hospital presentation were very similar (Table 2).

**Table 1:** Age and gender distribution of hospitalized patients with XDR^a^ typhoid and those with non-XDR typhoid in Hyderabad, Pakistan, from October 1, 2016, to September 30, 2018 (N=1452).

Characteristics			XDR typhoid cases (n=947), n (%)	Non-XDR typhoid cases (n=505), n (%)	All cases, n (%)
**Age (years)**					
	0-5		308 (33)	209 (42)	517 (36)
	6-10		236 (25)	134 (27)	370 (26)
	11-15		72 (8)	47 (9)	119 (8)
	16-25		142 (15)	43 (8)	185 (13)
	>25		189 (20)	72 (14)	120 (8)
**Gender**					
		Male	601 (54)	313 (62)	915 (63)
		Female	345 (45)	192 (38)	537 (37)

^a^XDR: extensively drug resistant; resistant to ampicillin, chloramphenicol, trimethoprim-sulfamethoxazole, ciprofloxacin, and ceftriaxone.

**Table 2:** Signs and symptoms of patients with XDR^a^ typhoid and those with non-XDR typhoid on hospital admission in Hyderabad, Pakistan, from October 1, 2016, to September 30, 2018 (N=1452).

Signs and symptoms	XDR typhoid cases (n=947), n (%)	Non-XDR typhoid cases (n=505), n (%)	All cases, n (%)
Fever (body temperature of <40°C	947 (100)	505 (100)	1452 (100)
Anorexia	947 (100)	505 (100)	1452 (100)
Headache	650 (68)	306 (60)	956 (65)
Abdominal pain	493 (52)	297 (58)	790 (54)
Diarrhea	488 (52)	297 (58)	785 (54)
Vomiting	385 (40)	196 (38)	581 (40)
Signs of anemia	360 (38)	89 (17)	449 (30)
Signs of toxicity	237 (25)	73 (14)	310 (21)
Constipation	210 (22)	71 (14)	281 (19)
Intestinal distension	218 (23)	72 (14)	290 (19)

^a^XDR: extensively drug resistant; resistant to ampicillin, chloramphenicol, trimethoprim-sulfamethoxazole, ciprofloxacin, and ceftriaxone.

The duration of illness between fever onset and hospitalization ranged 5-28 (median 20) days for patients with XDR typhoid, and 5-30 (median 13) days for those with non-XDR typhoid (*P*<.001). The conventional antibiotics taken before hospitalization among patients with XDR typhoid were ceftriaxone and ampicillin, whereas those among patients with non-XDR typhoid were cefixime, cephalosporin, and quinolones. In the hospitals, all patients with XDR typhoid were treated with a carbapenem or macrolides with a median hospitalization duration of 9.5 (range 2-25) days, while those with non-XDR typhoid were treated with cephalosporin and cefixime (n=483, 33%) and with ceftriaxone and ampicillin (n=22, 1.5%) with the median hospitalization duration of 6.5 (range 2-15) days.

Overall, ≥1 complications were reported in 360 (38%) patients with XDR and in 89 (18%) patients with non-XDR typhoid (*P*<.001). Ileal perforation was the most commonly reported complication in both patients with XDR (n=210, 23%) and those with non-XDR (n=71, 14%) typhoid (*P*<.001). Septicemia was the second-most commonly reported complication among both patients with XDR (n=97, 11%) and those with non-XDR (n=9, 1.7%) typhoid (Table 3).

The median duration of prehospitalization illness was longer among patients with complications (range 7-30 days, median 22 days) than among those without complications (range 5-25 days, median 6 days) (*P*<.001). Overall, mortality was documented in 17 of 947 (1.8%) patients with XDR Typhi infections and 3 of 505 (0.6%) patients with non-XDR Typhi infections (*P*=.06).

**Table 3:** Frequency of complications among hospitalized patients with XDR^a^ typhoid and those with non-XDR typhoid in Hyderabad, Pakistan, from October 1, 2016, to September 30, 2018 (N=1452).

Complications	XDR typhoid cases (n=947), n (%)	Non-XDR typhoid cases (n=505), n (%)	*P* value
Ileal perforation	210 (23.0)	71 (11.0)	<.001
Septicemia	91 (14.0)	9 (1.7)	<.001
Cholecystitis	27 (2.8)	4 (0.7)	.008
Pneumonia	10 (1.0)	1 (0.1)	<.001
Jaundice	13 (1.3)	1 (0.1)	.001

^a^XDR: extensively drug resistant; resistant to ampicillin, chloramphenicol, trimethoprim-sulfamethoxazole, ciprofloxacin, and ceftriaxone.

## Discussion

### Principal Findings

To our knowledge, this is the first study on the first major XDR typhoid outbreak worldwide and documenting mortality and morbidity in Pakistan. The delay in the initiation of appropriate antibiotic therapy is the leading cause of complications and deaths. XDR typhoid has reached alarmingly high levels, threatening to be potentially untreatable and leading to high morbidity and mortality in Hyderabad.

Our study reports a mean age was 15 years for patients with XDR typhoid and 12 years for those with non-XDR typhoid, the majority of our patients being male (n=915, 63%). In a study in Nepal, the mean age of patients with typhoid was 19 years, and 45% of them were male [15]. Typhi isolates from approximately two-thirds of cases (n=947, 66%) were XDR. A population-based study in 5 Asian countries reported that 23% of isolates were multidrug resistant [16].

Limited data on the case fatality rates of typhoid are available [17]. In this study, the case fatality rate was 1.8%. A study on the global burden of typhoid fever reported a case fatality rate of 1% [3].

Typhoid is an endemic disease in Pakistan. The major factors associated with its high prevalence rate include overcrowding, illiteracy, poverty, poor sanitation, and an unsafe supply of drinking water [18]. Early diagnosis and the use of appropriate antibiotics play a key role in reducing complications and deaths [19]. Poor sanitation and contaminated water supplies are serious infrastructure problems in Hyderabad, Karachi, and the rest of Sindh province. The 2 main cities of this province are affected by XDR Typhi. Public health officials predict that XDR Typhi will also cross borders and start spreading outside of Pakistan [20].

### Limitations

Our study has limitations, given its retrospective manner of conducting reviews of medical records of in-hospital patients; this has yielded limited information regarding prior treatments, including antibiotic use prior to hospitalization. Patients who were admitted to hospitals do not represent those among the general population of Pakistan.

### Conclusions

As this first XDR typhoid outbreak continues to spread, the increased duration of illness before hospitalization and increased rate of complications has important implications for clinical care and medical costs, and heightens the importance of prevention and control measures. To ensure that the minimization of delay in treatment due to the lack of proper diagnoses and access to hospitals, the establishment of certified laboratories and the recruitment of trained, well-informed physicians in geographically strategic areas will help in improving the situation. The provision of safe drinking water and proper sanitation is the ultimate solution to prevent future outbreaks. Setting up of surveillance for typhoid in the province and the whole country is important to determine whether XDR typhoid is limited to only these 2 cities or it has widely spread in Pakistan. Further prospective studies are also required for understanding the factors that are leading to the spread of XDR typhoid in Pakistan.

## Abbreviations

XDR: extensively drug resistant
